# Can e-learning improve the performance of undergraduate medical students in Clinical Microbiology examinations?

**DOI:** 10.1186/s12909-019-1843-0

**Published:** 2019-11-07

**Authors:** Niall T. Stevens, Killian Holmes, Rachel J. Grainger, Roisín Connolly, Anna-Rose Prior, Fidelma Fitzpatrick, Eoghan O’Neill, Fiona Boland, Teresa Pawlikowska, Hilary Humphreys

**Affiliations:** 1 Department of Clinical Microbiology, Royal College of Surgeons in Ireland, RCSI Education & Research Centre, Beaumont Hospital, Beaumont, Dublin 9, Ireland; 2 RCSI Information Technology Department, Royal College of Surgeons in Ireland, St. Stephen’s Green, Dublin 2, Ireland; 3 Department of Microbiology, Beaumont Hospital, Beaumont, Dublin 9, Ireland; 4 Department of Microbiology, Connolly Hospital, Blanchardstown, Dublin 15, Ireland; 5 Data Science Centre, Royal College of Surgeons in Ireland, Beaux Lane House, Lower Mercer Street, Dublin 2, Ireland; 6 Health Professions Education Centre, Royal College of Surgeons in Ireland, St. Stephen’s Green, Dublin 2, Ireland

**Keywords:** Clinical Microbiology, E-learning, Examination performance

## Abstract

**Background:**

Clinical Microbiology is a core subject in medical undergraduate curricula. However, students struggle to cover the content and clinically contextualise basic microbiology. Our aim was to evaluate student engagement with new e-learning material and to investigate the impact it had on examination performance in a Clinical Microbiology module.

**Methods:**

An online resource was designed to support didactic teaching in a Fundamentals of Clinical Microbiology module. One cohort of students had access to the online material (2017/2018 class) and the other did not (2016/2017 class). Each cohort sat the same multiple-choice question (MCQ) and short-note question (SNQ) examination papers and the impact of engagement with the online resource and examination performance was analysed.

**Results:**

Both groups were of the same academic standard prior to beginning the module. In the 2017/2018 cohort, 227/309 (73.5%) students had ≥80% engagement with the content. Students engaged most with the index of pathogens and pathogen focused clinical cases related to diverse genera and families of clinically important microorganisms. A statistically higher difference in the mean percentage grade in both the MCQ and SNQ examinations was seen for 2017/2018 compared to 2016/2017 cohort. For the MCQ examination, the 2017/2018 cohort were on average 5.57% (95% confidence interval (CI): 3.92 to 7.24%; *P* < 0.001) higher, and for the SNQ examination the 2017/2018 cohort were on average 2.08% (95% CI: 0.74 to 3.41%; *P* = 0.02) higher. When the results were adjusted for previous examination performance, for every percentage increase in online engagement the grade in the SNQ examination only increased by 0.05% (95% CI: 0.02 to 0.08) on average.

**Conclusions:**

These findings suggest students engage with e-learning when studying and that such activities may help students perform better in assessments.

## Background

Clinical Microbiology is an essential component of all medical curricula [1, 2]. As a biomedical discipline and pathology sub-speciality, it has multiple facets with undergraduate medical students requiring an understanding of bacteriology, virology, mycology, pathogenesis of infection, laboratory diagnostics, the pharmacology and therapeutic uses and stewardship of antimicrobials, public health and epidemiology, infection prevention and control and other preventative measures, such as immunisation. A recent survey of 104 United States medical school curricula revealed that most often, pre-clinical medical students receive a single block of microbiology teaching either alone or concurrently with another courses [1]. The rationale for this approach is rooted in a more traditional approach to curriculum design where the fundamentals of microbiology would be taught so that the students can apply this knowledge to complex clinical scenarios that may arise from infection in practice. These modules also mainly consist of lectures, tutorials and other face-to-face modes of teaching [1]. As a result, introductory modules covering the fundamentals of Clinical Microbiology are often content heavy and students frequently struggle to cover the breadth in detail, which can often lead to unwanted stress amongst early career students [2, 3]. In addition, students can struggle to see the clinical relevance of the fundamentals of the biomedical sciences. This, with an over-reliance on large group lectures without clinical context encourages learning compartmentalisation and in the long-term the loss of the basic knowledge [4]. Our own experiences also suggest that students struggle to see the clinical relevance of the fundamental aspects of the microbiology. For example, students may be taught about the many toxins of *Staphylococcus aureus* but many may struggle to recognise the role of the toxins in the pathogenesis and the presentation of the infection.

Technology-enhanced learning (TEL) and online- or e-learning approaches are very popular in health professions education and they are often received positively by students who all have access to portable electronic devices and computers [5, 6]. This means students can study freely, in their own time and at their own pace. Online material used effectively can also provide a means of self-assessment with students having the ability to identify gaps in their knowledge and receive feed-back. However, the use of e-learning and its benefits should always be scrutinised [7] Some also argue that care needs to be taken when using e-learning and technology in medical education to avoid it being seen as a substitute for hands-on training of clinical skills [8]. Furthermore, whether online learning improves retention of knowledge and improves exam performance is still debatable.

In 2017, an online module was created to compliment the didactic teaching of a foundation module in Clinical Microbiology that is delivered to second year undergraduate medical students at the Royal College of Surgeons in Ireland (RCSI). The module was designed to contextualise basic microbiology within a clinical scenario. The aim of this study was to determine if students engage with online learning when studying and to evaluate the impact of this approach on exam performance.

## Methods

### Study design, student population and ethical approval

This was a comparative study to evaluate the impact of a new online educational intervention on examination performance. Information on second year undergraduate medical students at the RCSI in the academic years of 2016/2017 and 2017/2018 was used. There were 329 students in 2016/2017 cohort and 334 students in 2017/2018 cohort. However, only 313/329 (93.7%) in the 2016/2017 cohort and 309/334 (92.5%) sat the end-of-semester examination for the module and hence were included. Ethical approval was given from the RCSI Research Ethics Committee to extract overall exam results for each student only. The need for formal consent from students to collect this information was not required.

### Module structure

The Foundations in Microbiology (FIM) module, is the first module in the second semester of year two, carries 10 European Credit Transfer and Accumulation System (ECTS) credits and is 5 weeks long in a 12 week semester. There are 29 lectures and four tutorials covering the basic bacteriology of 14 genera of clinically important bacteria, the basic virology and four families of clinically important viruses, and the basic mycology of clinically important fungi. In addition, students learn the principles of antibiotic stewardship, the pharmacology of antimicrobials, immunisation and infection prevention and control. The module learning outcomes are as follows;

1. Describe the characteristics of the major groups of medically important bacteria, viruses, fungi and other microorganisms
2. Relate the basic physiological & molecular features of these microbes to the practice of medicine
3. Explain how microbes cause infection & disease i.e. pathogenicity and spread
4. Identify the characteristics of common pathogens
5. Apply the basic principles of antibiotic chemotherapy & use antibiotics correctly
6. Practice the principles of infection prevention, e.g. vaccination, hand hygiene
7. Apply the principles of laboratory diagnosis & use the laboratory intelligently

### Content design

**Fig. 1:**
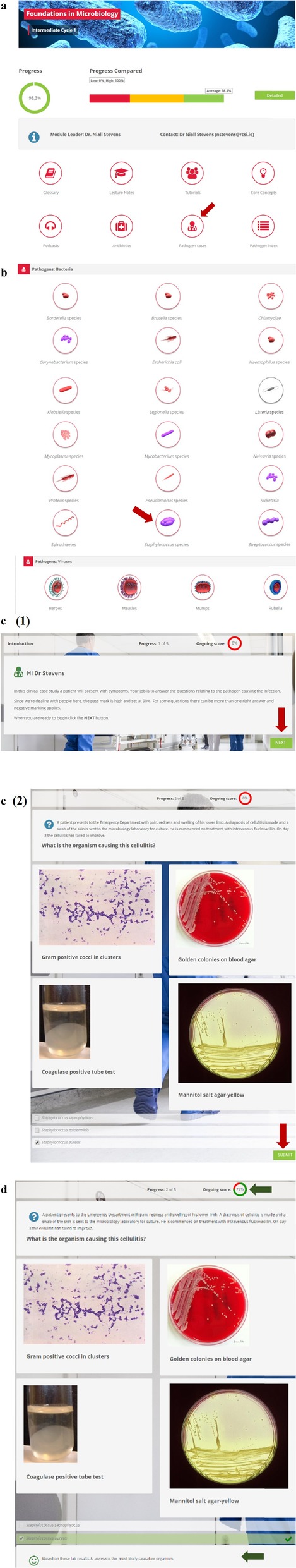
Format of the clinically important pathogen cases of the online content. **a** All students had the same home-page through which they could access the different components of the online material. **b** The basic microbiology for 18 clinically important genera of bacteria, as well as clinically important viruses and fungi (not shown) were covered. Red arrows indicate how the student accessed each activity. **c (1) & (2)** The interactive activities framed within the context of a clinical scenario with many containing laboratory findings or other clinical data. Students had unlimited attempts to complete each activity. **d** Students received feedback after each attempt and could track their progress (green arrows)

The online content was designed to align with the face-to-face teaching and to address the overall modular learning outcomes listed above. The content consisted of an alphabetical glossary of important terms in Clinical Microbiology and question/answer-type activities on core concepts, such as cell morphology and prokaryotic cell structure, microbial growth and physiology, bacterial genetics, virology, mycology, and pathogenesis of infection. There were also podcasts with accompanying multiple choice question (MCQ)-style quizzes on difficult subjects or important topics in Clinical Microbiology and question/answer-type activities on important concepts relating to antimicrobials (Table 1). Clinical cases scenarios were written for each important genera or family of microorganisms to create question/answer-type activities with feedback and to provide the students with a clinical context to the basic microbiology e.g. virulence, pathogenesis of infection, specimen collection and laboratory identification (Fig. 1). Each pathogen name was hyper-linked to an alphabetical index. Each index entry summarised the epidemiology, virulence, pathogenesis of common infections caused, the laboratory diagnosis, and aspects of antimicrobial management and prevention. Some clinically important pathogens e.g. influenza were dealt with in other system-based modules. Requirements relating to completion of the online material were outlined in the Marks and Standards document for this specific academic year and students were also given clear instructions of what was expected in a face-to-face introductory teaching session that gave an overview of the module. Students were expected to complete all components of the online content but there were no academic consequences for not engaging. Students accessed the material via the virtual learning environment, Moodle. Students could track their progress as they completed each aspect of the online activities and they could compare this progress to that of the entire class.

**Table 1:** List of online activities under core concepts, podcasts and antibiotics

Activity Title	
Core Concepts	
Classification of microorganisms	
Bacterial morphology & cell structure	
Bacterial growth & physiology	
Pathogenesis of bacterial infections	
Introduction to virology	
Introduction to mycology	
Appropriate use of the microbiology diagnostic laboratory	
Introduction to healthcare-associated infections	
Introduction to opportunistic infections	
Podcasts & MCQ quizzes	
Bacterial genetics	
Healthcare-associated infections	
Streptococci	
Pathogenesis of viral infections	
Cytomegaloviruses	
Measles virus	
Cell wall active antibiotics: penicillins & cephalosporins	
The aminoglycosides, quinolones and macrolides	
Antibiotics	
Classification of antibiotics	
Antibiotic stewardship	
Mode of action of antibiotics	
Important antibiotic resistant microorganisms	
Mechanisms of antibiotic resistance	
Adverse effects of antibiotics

### Approach to assessment

To progress, students must pass a summative end-of-semester assessment that consists of MCQs and constructed response items in the form of short notes questions (SNQs). The MCQ assessment requires the students to interpret clinical and laboratory data relating to ten different pathogens and the infections they cause. The students must answer 30 MCQs (three MCQs per pathogen and infection scenario). On the SNQ examination paper, the students must answer all five questions. All assessments were blue-printed to ensure alignment to learning outcomes, stage appropriateness and to ensure the breadth of the course was being examined. All papers were reviewed by an external examiner prior to the assessment. SNQs had individual and detailed model answers and all were accompanied by a marking scheme (0–100%) consisting of eight grades divisions each with a detailed descriptor for the examiner. All SNQs were double-marked blind by two different examiners and the marks were adjudicated by a third. Both the 2016/2017 and 2017/2018 cohorts of students sat the same MCQ and SNQ-papers so that a direct comparison in grades could be made.

### Assessment of online material on examination performance

Exam results were collected for students who completed year two in the academic year 2016/2017, and for those who completed it in the 2017/2018 academic year. Additionally, each student’s previous end of semester result i.e. year 2, semester 1 overall grade was collected as a proxy for the student’s overall academic standard. The 2017/2018 cohort of students were provided with online material. The 2016/2017 cohort had no additional online material. For each student in the 2017/2018 cohort, the percentage of online material viewed was calculated as a measure of engagement with the online material.

### Statistical analysis

Engagement in online material in the 2017/2018 cohort was initially explored. This was calculated as the amount of available online material that was accessed at least once by each student out of the total amount of material available. This was converted to a percentage and also categorised as follows: < 20, 20- < 40%, 40- < 60%, 60- < 80% and ≥ 80% to explore visually.

Students’ previous exam grades (2016/2017 vs 2017/2018 cohort) were explored using a t-test to compare baseline differences between the cohorts. Following availability of online material for the 2017/2018 cohort, average MCQ and SNQ scores for the two cohorts were compared. Furthermore, regression analysis using the 2017/2018 cohort only, was explored to assess the relationship between engagement (as a continuous variable) and final exam grade, adjusting for previous exam performance.

## Results

### Overall engagement with the online content

**Fig. 2:**
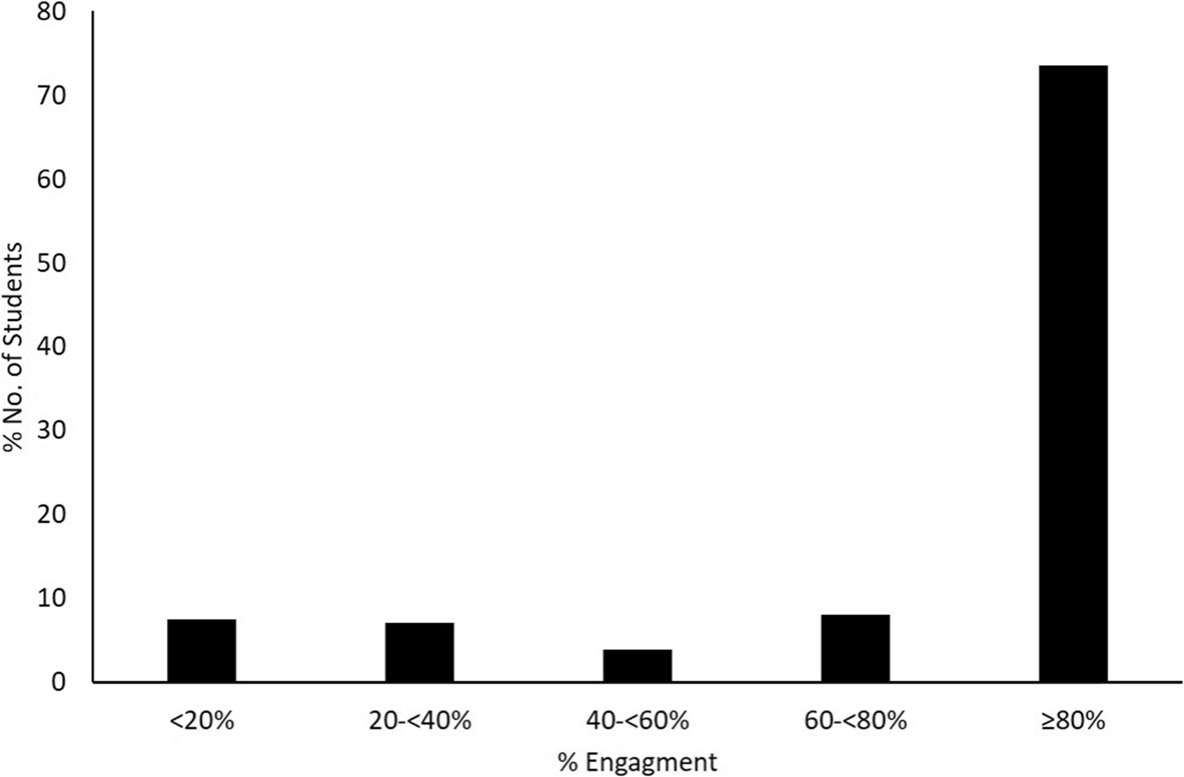
Engagement of 2017/2018 cohort of students with online content**.** This student cohort had 309 students, all of which had access to the online material for the entire 12 week semester. Of these, 7.44% (23/309) had < 20% engagement, 7.12% (22/309) had between 20- < 40% engagement, 3.88% (12/309) had between 40- < 60% engagement, 8.09% (25/309) had between 60- < 80% engagement and 73.46% (227/309) had ≥80% engagement

All 309 students who sat the end-of-semester examinations in the 2017/2018 cohort engaged with the online content at some point over the entire 12 week semester. However, the level of engagement varied from a low of 23/309 students (7.4%) having < 20% engagement to a high of 227/309 students (73.5%) having ≥80% engagement with the online content (Fig. 2). The top three most viewed components of the online content are listed in Table 2. Students accessed online content related to healthcare-associated infections (HCAIs) most with the activity that covers core concepts related to this topic being accessed 71,737 times and the podcast on the same topic being accessed 12,340 times. The top three pathogen-focused clinical cases related to herpes virus infection, community-acquired pneumonia caused by *Streptococcus pneumoniae* and pharyngitis caused by *Streptococcus pyogenes* (group A streptococcus). The glossary of terms was accessed the least by the students. Students also only accessed the four animated videos related to the mode of action of important class of antibiotics on average 325 times (range: 299 times to 396 times). The pathogen index was accessed by the majority of registered students with 329/334 (98.5%) using this component of the online material at some point in time.

**Table 2:** The most accessed online activities under each individual subject

Online Activity	No. of times accessed^a^	No. of individual users/registered users (%)^b^
Core Concepts		
Bacterial morphology & cell structure	74,959	280/334 (83.8)
HCAIs & Infection prevention & Control	71,737	262/334 (78.4)
Bacterial genetics	52,706	267/334 (79.9)
Podcasts		
HCAIs	12,340	281/334 (84.1)
Aminoglycosides, quinolones & macrolides	11,648	275/334 (82.3)
Streptococci	10,953	288/334 (86.2)
Antibiotics		
Important resistant bacteria	110,964	279/334 (83.5)
Adverse effects of antibiotics	39,617	267/334 (79.9)
Classification of antibiotics	31,623	307/334 (91.9)
Pathogen focused online cases		
Herpes virus infections (Herpes simplex & Varicella zoster)	28,521	288/334 (86.2)
*Streptococcus* spp.: A 68 year-old male with CAP	26,819	286/334 (85.6)
*Streptococcus* spp.: A 7 year-old female with pharyngitis		
Pathogen Index	8644	329/334 (98.5)
Glossary of terms	1457	288/334 (86.2)

^a^Could be accessed more than once by each registered student

^b^Not all registered users sat the end-of-semester examination

### Impact of online learning on examination performance

Before determining the impact of the online content on examination performance in the FIM end-of-semester assessments, the academic standard of both cohorts of students i.e. those with access to the material (2017/2018 class) and those without access (2016/2017 class), was determined by comparing their overall grades from the previous year. There was no evidence of a difference between the overall average grade achieved by the 2017/2018 class and the 2016/2017 class with a mean grade difference of 0.24 (95% CI:-1.18 to 1.66; *P* = 0.742) being observed.

When the impact of the online material on examination performance in both the MCQ and SNQ written paper was assessed, a statistically higher difference in the mean percentage grade for the 2017/2018 cohort compared to 2016/2017 cohort was seen. For the MCQ examination, the average difference was 5.57% (95% CI: 3.92 to 7.24%; *P* < 0.001), and for the SNQ examination, 2.08% (95% CI: 0.74 to 3.41%; *P* = 0.02).

Looking at the 2017/2018 cohort only and exploring engagement with online material, while adjusting for students’ examination performance in the previous year, evidence of a significant relationship between the level of engagement with the online content and the grade received in the SNQ examination can be seen. For every percentage increase in online engagement, the SNQ score increased on average by 0.05% (95% CI: 0.02 to 0.08%, *P* = 0.002). There was no evidence of significant relationship between engagement with the online material and performance in the MCQ examination (0.03, 95% CI: − 0.04 to 0.07%, *P* = 0.179).

## Discussion

The findings of this study indicate that student engagement with the new online content was high with over 70% of the class having > 80% engagement. The data also suggests that having access to the online content helped the 2017/2018 cohort to perform better in the end of semester examinations. This was most evident in the SNQ component of the assessment.

E-learning has been shown to have a positive impact on the achievement of learning outcomes in health professions education. A recent study found that a mixed methods approach that combined traditional learning activities with e-learning improved clinical skills in nurses [9]. Another study examining the impact of e-learning on achievement of learning outcomes in a medical immunology course found that on average 3.6% of the class performed better on the immunology component of the examination, which was linked to the level of engagement with the online material [10]. While the statistical analysis indicated that greater engagement with the online content resulted in a higher grade in the SNQ examination, a relationship between greater engagement and better exam performance in the MCQ component could not be found in our study. When well-constructed, MCQs are useful for assessing knowledge in courses with a lot of content, such as our introductory module. However, MCQs focus on a single piece of content and it be can be especially challenging for students to make the correct decision when answering if the difference between a right and a wrong answer is extremely nuanced. Students may also adopt a surface learning approach when preparing for MCQ papers of content heavy courses, which has been shown to have a negative impact on academic achievement [11]. Often there is much debate in health professions education over the benefits of one form of assessment over another e.g. MCQs *versus* SNQs. However, a recent study has shown that MCQs and SNQs are equally as effective at assessing higher order skills once there are no flawed MCQs [12].

Overall, engagement with the pathogen focused online cases was high, with students engaging most with those that focused on diverse groups of pathogens such as the herpes viruses and streptococci. Given the number of clinically important pathogens in these groups and the variety of clinical presentations that can arise from infection, it is not surprising that students accessed the related material the most. Providing context is essential for medical students if they are to understand the pathogenesis of infections and the role of the pathogen, how a laboratory and clinical diagnosis is made and how the patient is managed. Modern medical curricula often have an integrated, blended and student-centred approach to interest students, to give them the required breadth of knowledge to understand clinical scenarios and to also accommodate the different ways in which students approach their learning [13–15]. Case-based learning (CBL), whether it be face-to-face or online, is a well-recognised, and beneficial, approach and it has been used extensively when teaching health-profession students [16, 17]. More specifically, with CBL the use of technology and other online approaches to teaching students about Clinical Microbiology and infection have become more prominent in recent years with audience response devices, videos, online cases, virtual patients and gaming being used [5, 6, 18].

While these findings suggest that increased engagement overall with the online material resulted in better performance in the examinations, a direct link between engagement with specific content and performance in the related question on the MCQ or the SNQ papers cannot be made, which is a limitation of the study. Also, no other influences on student engagement could be determined as no other demographics could be collected. Another limitation is that only 1 year, post introduction of the online content, was examined. However, a strength of the study lies in the direct comparison in exam performance that could be made. Both cohorts of students sat the exact same examinations in sequential years, which would not have been possible if a third cohort was included as the integrity of assessment process may have been compromised if students recognised the similarities in papers. While there is a possibility that the 2016/2017 conveyed details of the assessment to the next year coming, we have no evidence to suggest this occurred. There is also a robust exam-setting procedure within our institution that ensures the integrity of the papers are maintained and our students do not have access to a bank of past papers that would allow for predictions to be made based on previous assessments. We therefore believe any possible influence of the 2016/2017 cohort of students on the examination performance the 2017/2018 cohort to be minimal, if at all present.

Facilitating the varied approaches to learning and study is essential to ensuring the needs of each student are being accommodated. TEL is becoming more prominent in health professions education as it can be used to create more student-centred curricula while optimising lesson designs to make them more engaging [19]. Several studies have examined the use of TEL across the spectrum of health professions disciplines with many noting varied impacts on the students’ experience [20–23]. Often, the introduction of novel approaches to learning, including online approaches, are received well by students but their impact on retention of knowledge can be difficult to ascertain. One study examining the use of e-modules in the education of paediatric medical students did not find any improvement in the scores of National Board of Medical Examiners paediatric examinations following their introduction [20]. Moreover, Goodchild (2018) highlights the need for critical appraisal on the use of TEL in nursing education especially as it becomes more prominent in related curricula. The author suggests that nursing academics should reflect on the impact the introduction of technology has had by examining what has been lost and weighing it against what has been improved upon [24].

## Conclusions

Our findings suggest that e-learning in an introductory course to Clinical Microbiology is well received and can have a positive impact on examination performance. Supporting didactic teaching on clinically important pathogens, laboratory diagnosis and antimicrobial prescribing and pharmacology is important as these areas can often be content heavy and difficult to study. Indeed, a recent study found that e-learning can positively improve the prescribing skills of medical students under examination conditions [25]. As the curricula of health professional courses become more integrated, content heavy subjects, such as Clinical Microbiology, are likely to be scrutinised in order to reduce cognitive overload and improve the student experience. TEL, when used effectively, could ensure this important clinical discipline maintains its prominence within these newly designed curricula and help students maximise their learning potential and perform effectively in related assessments.

## Data Availability

The datasets used and/or analysed during the current study are available from the corresponding author on reasonable request.

## Abbreviations

CBL: Case-based learning
ECTS: European Credit Transfer and Accumulation System
FIM: Foundations in Microbiology
HCAIs: healthcare-associated infections
MCQ: Multiple-choice question
RCSI: Royal College of Surgeons in Ireland
SNQ: Short note question
TEL: Technology-enhanced learning
